# Effects of Ozone Treatment on Microbial Status and the Contents of Selected Bioactive Compounds in *Origanum majorana* L. Plants

**DOI:** 10.3390/plants9121637

**Published:** 2020-11-24

**Authors:** Natalia Matłok, Tomasz Piechowiak, Miłosz Zardzewiały, Józef Gorzelany, Maciej Balawejder

**Affiliations:** 1 Department of Food and Agriculture Production Engineering, University of Rzeszow, St. Zelwerowicza 4, 35-601 Rzeszów, Poland; mzardzewialy@ur.edu.pl (M.Z.); gorzelan@ur.edu.pl (J.G.); 2 Department of Chemistry and Food Toxicology, University of Rzeszow, St. Ćwiklińskiej 1a, 35-601 Rzeszów, Poland; tomek.piechowiak92@gmail.com (T.P.); maciejb@ur.edu.pl (M.B.)

**Keywords:** herbs, ozone, fumigation, microbial load, antioxidant potential, polyphenols, HS-SPME

## Abstract

This study presents the effects of ozone treatment on microbial status and contents of selected bioactive compounds in marjoram plants. *Origanum majorana* L. is a widely used plant which in the course of production is affected by microbial infections. One of the ways to reduce microbial load involves application of a strong oxidant, such as ozone. In order to determine the effects of ozonation, a number of analyses were carried out including microbiological tests (aerobic colony count, yeast and mould count, and mesophilic lactic acid bacteria count) and chemical tests assessing total antioxidant potential, total polyphenols, and volatile fraction composition. Ultimately, the findings showed considerable (6-log) reduction in microbial load, with unchanged composition of headspace volatile compounds. Furthermore, the raw material obtained presented elevated the contents of the selected bioactive compounds. It was shown that the most beneficial effects are achieved when ozone treatment is applied at a rate of 1 ppm for a duration of 10 min.

## 1. Introduction

Marjoram is a medicinal plant, and it is used as a seasoning [1]. It is most widely known in the form of crushed, grey and green herbal flakes with a unique scent. The most common form of this spice is marjoram flakes, while complete sections of dried plants are rarely offered commercially. Marjoram (*Origanum Majorana* L. *syn. Majorana hortensis*) is classified in the mint family (*Lamiaceae*) and is native to the Mediterranean Region, North Africa, and Western India, where the plants may achieve up to one metre in height. Currently, it is grown in many countries in Asia, North Africa, and Europe, mainly in India [2]. It is also widespread in the temperate regions of the Himalayas from Kashmir to Sikkim at an altitude of 500 to 1200 m above sea level. [3,4]. In their natural environment, the plants are frequently infected by microorganisms which are rare in other parts of the world, particularly in Northern Europe [2]. The source of contamination with microorganisms in herbal raw material (bacteria or fungi) is epiphytic flora and flora from the environment, i.e., soil, water, or air. Research shows that the raw material from cultivation is more contaminated with pathogenic microorganisms than that collected in nature [5,6]. During cultivation, marjoram plants are also infected by many species of fungi and bacteria, causing some damage to crops and influencing the quality of the final raw material. The presence of pathogenic microorganisms causing diseases of herbs is a real cause of microbial contamination of plant material. The quality of these raw materials is significantly reduced by secondary metabolites (mycotoxins) produced by pathogenic fungi on plants during their cultivation or storage. Contamination of the raw material of herbal plants by bacteria is much less common [7,8,9]. The main component of marjoram is essential oil, which is used in various applications [10]. According to Góra and Lis [11], its content in fresh plant material amounts to 0.3–0.9% and in dry material amounts to 0.7–3.5%. Marjoram oil is known for its antiseptic, dehydrating, anti-inflammatory, and anti-acne activities. As regards its antimicrobial activity, it is as effective as cinnamon and clove oil [12]. Given their diverse chemical composition, physicochemical parameters, and scents, marjoram oils are classified as various chemotypes. Other active substances contained in the raw plant material include tannins, reducing sugars, vitamins, flavonoids, phytosterols, pectins, phenolic acids, bitter compounds, organic acids, and mineral salts [5,11,13]. According to Suhaj [14], marjoram contains antioxidants such as carvacrol, eugenol, polyphenols, ascorbic acid, ursolic acid, and oleanolic acid. A study by Newerli-Guz [15] showed that the levels of total phenols in marjoram are varied depending on the place of origin. It was found that marjoram from Egypt contained 28.41–98.43 mg GAE g^−1^, compared to marjoram grown in Poland in which the related value was in the range of 26.66–54.64 mg GAE g^−1^. Duletić-Laušević et al. [16] examined the contents of phenolic compounds in marjoram originating from Serbia, Greece, Egypt, and Libya. They found that the raw material from each of these countries differed in the contents of rosmarinic, caffeic, and chlorogenic acids as well as arbutin and luteolin-7-O-glucoside. Fecka and Turek [17] investigated the levels of polyphenols in raw marjoram materials and found that its mean content amounted to 47.92 mg g^−1^.

The quality of plant material, including marjoram, depends on a number of factors, such as soil, climate, as well as agricultural engineering techniques applied in the production process. There are also other abiotic factors which affect the chemical composition of crops. One of them is ozone. As reported by Matłok et al. [18], ozone may affect the contents of bioactive compounds in pine shoots subjected to ozone treatment. This research showed that ozone treatment carried out in specific conditions produced an increase in polyphenols content, total antioxidant potential, and vitamin C. The findings also showed that the material was also rich in essential oils, which potentially may be sensitive to the oxidising activity of ozone. The study also demonstrated that pine shoots subjected to a correctly designed ozonation process retain the same content of oil and that its composition is unchanged. Ozone also impacts microbiological composition of the plant material. As demonstrated by Piechowiak et al. [19], the ozonation process makes it possible to significantly reduce microbial load while retaining the fine quality of the plant material. This is particularly important in the case of decontamination of plants grown in tropical climates as they are a frequent source of diseases brought to other climate zones. 

The aim of the study was to examine the effectiveness of ozone treatment on reducing microbial load in marjoram plants. Effectiveness of the process was assessed using microbiological analysis and chemical assays designed to determine chemical composition of the raw plant material. 

## 2. Results and Discussion

### 2.1. Antioxidant Activity and Bioactive Compounds Content 

**Figure 1:**
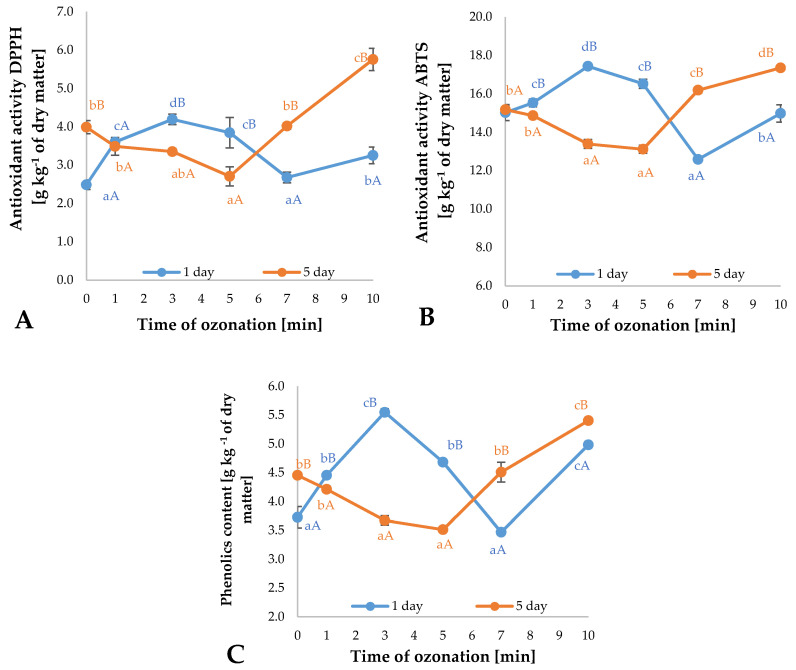
Antioxidant activities (DPPH (2,2-difenylo-1-pikrylohydrazyl) test (**A**), ABTS (2,2′-azynobis-(3-etylobenzotiazolino-6-sulfonian) test (**B**) and total polyphenolic content (**C**) in *Origanum majorana* L. leaves depending on the time ozonation (n = 3): differences in results between the time of ozonation in current days are indicated by different small letters, and difference between terms of measurements are indicated by different capital letters; difference is noted at significant level *p* < 0.05.

Ozone is one of the abiotic elicitors impacting the contents of bioactive compounds in raw plant materials. The use of gaseous ozone in the fumigation process applied to marjoram plants resulted in increased antioxidant potential of the raw material (Figure 1A,B). A significant increase in the contents of antioxidants, measured using ABTS (2,2′-azynobis-(3-etylobenzotiazolino-6-sulfonian)) and DPPH (2,2-difenylo-1-pikrylohydrazyl) methods, in comparison to the plants in the control variant was identified on the first day following ozone treatment in the plants exposed to ozone (1 ppm concentration) for 1, 3, 5, and 10 min. However, compared to the control sample, the highest increase was identified in the case of plants subjected to ozonation for 3 min. Conversely, on the fifth day following ozone treatment, an increase in antioxidant potential in the marjoram plants, compared to the control, corresponded only to longer duration of exposition, amounting to 7 and 10 min. This may have been associated with ozone penetration through the open stomata of the marjoram plants and the reaction with components of calcium ion channels in the plasma membrane. It is likely that this reaction resulted in penetration of cell membranes, which induced intensified production of reactive oxygen species (ROS) and certain enzymes, such as superoxide dismutase and peroxidases. Increased activity of these enzymes led to controlled decomposition of ROS through increase in concentrations of low-molecular-weight antioxidants [20,21]. Increased antioxidant potential of raw plant materials exposed to gaseous ozone was also reported by other researchers who additionally observed that sensitivity to ozone differs depending on the plant variety and species as well as the method applied (ozone concentration and duration of the treatment). It is very difficult to compare the effects, particularly in a situation when different conditions and different varieties of a given species were used. Moreover, attempts to assess effectiveness of ozonation are additionally hampered by the fact that some researchers do not present detailed information about the conditions, variations, and varieties [22]. Matłok et al. [23] performed fumigation of red-veined sorrel plants with 1 ppm ozone and identified significant changes in antioxidant potential on the first and fourth days following treatment. The best effects on the first day following treatment, i.e., 18.9% (ABTS method) and 24.7% (DPPH method) increases in antioxidant potential compared to the control, were identified in the specimens exposed to ozone for 10 min. 

Fumigation of marjoram plants, based on the design presented here, also impacted the total contents of polyphenols in the materials (Figure 1C). Application of gaseous ozone at concentrations of 1 ppm for durations of 1, 3, and 5 min produced significant increases in total polyphenols on the first day following treatment compared to the related values in the control specimens (not subjected to ozonation). It is likely that the increase in the contents of polyphenols in the plants was associated with activation of enzymes involved in biosynthesis of polyphenols mediated by ozone and/or with accumulation of reactive oxygen species in response to abiotic stress. Piechowiak and Balawejder [24] reported that, when raspberries were stored in atmosphere periodically treated with ozone, there was a rapid increase in the activity of phenylalanine ammonia-lyase—the first enzyme in the phenylpropanoid pathway leading to production of polyphenols. Increase in the total contents of polyphenols as a result of gaseous fumigation applied to plants was also reported by Matłok et al. [23]. The researchers demonstrated that, by correctly defining dose of ozone during the fumigation of red-veined sorrel plants applied at a concentration of 1 ppm, it is possible to increase the contents of polyphenols in raw material. Gutiérrez et al. [25] reported that ozone treatment applied for 10 min to Rocket plant (*Eruca sativa Mill*.) at a rate of 1 and 10 ppm results in increased contents of polyphenols in the material. 

### 2.2. Chemical Composition of HS-SPME

**Figure 2:**
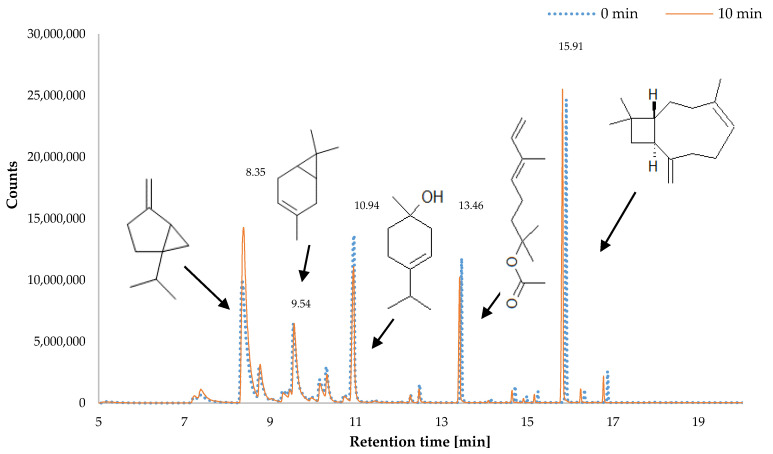
Chromatograms SPME-GC (Head Space–Solid Phase Microextraction coupled with Gas Chromatography) for the volatile fraction of leaves of *Origanum majorana* L. leaves depending on the time ozonation in one day from ozonation (n = 3).

The ozone treatment applied to marjoram plants produced a change in the profile of aromatic compounds (Table 1 and Table 2). On the first day following treatment, a correlation was observed between the contents of sabinene and duration of exposure to ozone. The compound is a typical example of a highly volatile monoterpene, which means that it may determine the perceived scent, directly affecting the quality of the raw material. Its increased biosynthesis is associated with overall activation of the enzymatic apparatus in the marjoram plants. As a typical monoterpene, it is synthesised in accordance with the isoprene rule through condensation from precursors such as isopentenyl pyrophosphate (IPP) with dimethylallyl pyrophosphate (DMAPP) [26]. Linear monoterpenes formed from the precursors undergo a process of cyclizing, which is induced by various reactions. The cyclisation may be promoted by free-radical reactions induced by ROS. At the same time, there was a decrease in the contents of oxygen derivatives such as 1-terpineol. Oxygen derivatives are mainly formed in the process of hydrocarbon metabolism. Hence, it is likely that the metabolism was restricted due to the disrupted biochemical balance of the plants exposed to ozone. This hypothesis is supported by composition of the volatile compounds on the fifth day after the treatment (Table 2). On that day, the differences in the composition of the volatile compounds between the plants exposed to ozone and the control specimens were no longer clear. During this period, the plants returned to the state of biochemical balance preceding ozone treatment. Possibly, enzymatic activity in the samples was levelled, which means the biosynthesis of the volatile compounds, as secondary metabolites, was the same in the control specimens and in the samples exposed to ozone (Figure 2, Table 2). This is important from the viewpoint of crop production because the plants treated with ozone should be designated for processing or sale at the latest on the fifth day, as later on, they lose their properties associated with the elicitation process. 

### 2.3. Microbial Load in the Raw Material

The microbiological analyses taking into account *Origanum majorana* L. plants subjected to ozone treatment showed the effect of ozone on the total count of aerobic bacteria, yeast, and mould, as well as mesophilic lactic acid bacteria (Table 3). Fumigation of marjoram plants with gaseous ozone applied at a rate of 1 ppm produced a significant decrease in aerobic colony count as well as yeast and mould count (cfu g^−1^) on the first and fifth days following treatment. The best result was observed when marjoram plants were exposed to ozone for 10 min. In this case, the counts of aerobic bacteria on the first and fifth days following treatment were reduced by 3 and 2 log cfu g^−1^ relative to the control (no ozonation). A similar effect was observed on the fifth day after the ozonation process. The decrease in aerobic colony count in the marjoram plants treated with ozone was associated with the antibacterial activity of ozone which results from its oxidising ability. Ozone, which in this respect is only inferior to fluorine and peroxysulfate, is one of the strongest oxidants (Eo = + 2.076 V) [27]. The effect of ozone on decrease in microbial stress in raw plant materials was also shown by other researchers. Piechowiak et al. [24] applied gaseous ozone at a rate of 8–10 ppm for 30 min (every 12 h) to improve storage life of raspberries in room temperature, and 48 h after the ozone treatment, they observed a reduction in the total count of mesophilic aerobic bacteria by 1.18 log cfu g^−1^ compared to the control. On the other hand, Torlak et al. [28] found that ozone treatment applied to dried oregano for 120 min (with ozone concentrations of 2.8 and 5.3 mg L^−1^) produced significant reductions of 2.7 and 1.8 log in an aerobic colony count. Kazi et al. [29] reported reductions in aerobic bacteria by 4 log in the case of dried oregano and by only 1–2 log cfu g^−1^ in the case of other herbs following 30 or 60 min of gaseous ozone treatment applied at a rate of 4 ppm. Ozone treatment applied to the marjoram plants produced a decrease in yeast and mould count in the material, while on average, plant materials contain 10^1^–10^6^ colony forming units per 1 gram [30]. Both on the first and the fifth days following the ozone treatment, the best results were identified in plants exposed to ozone for 10 min; in this case, yeast and mould counts in both measurements were reduced by 2 log cfu g^−1^ compared to the control. A reduction in yeast and mould count as a result of ozone treatment applied at a rate of 4 ± 0.5 ppm to tomatoes in storage was reported by Aguayo et al. [31]. They found that cyclic exposure to ozone (every three hours) reduces the count of mesophilic aerobic bacteria by 1.1 log cfu g^−1^ and fungi count by 1.75 log cfu g^−1^ in the relevant material compared to control specimens (no ozone exposure). The applied ozone treatment scheme, irrespective of the duration of exposure, also led to a reduction in the count of mesophilic lactic acid bacteria to a value below a detection threshold. 

## 3. Materials and Methods 

### 3.1. Pot Experimental Design

The plants of *Origanum majorana* L. were produced in course of a pot experiment, conducted in a greenhouse, and by using sterile peat medium (pH in KCl, 6.4; P_2_O_5_, 40.4; K_2_O, 244.2; Mg, 60.0; and N total, 0.74%). Mineral fertilisation of the soil was applied as described by Matlok et al. [32]. The experiment was carried out in three replications, each comprising an area of 0.5 m^2^ and 32 nursery pots with a volume of 0.78 dm^3^. In each pot, 20 seeds were planted at a depth of approx. 0.5 cm. Moisture of the medium in which marjoram was grown was maintained at a level of 60% of field water capacity. The production conditions, such as temperature and air humidity, were controlled. LED-based technology for lighting of plants during cultivation was utilised (photoperiod used: 12/12 h day/night). Throughout the duration of the experiment, the temperature on average amounted to 25 °C. Controlling these conditions made it possible to produce raw material with a homogeneous microbial load. No crop protection chemicals were applied during the experiment.

### 3.2. Determination of Ozonation Process Phytotoxicity 

Methodologies described in detail in the Appendix C were followed by determination of ozonation process phytotoxicity.

### 3.3. Ozone Treatment of the Plant Material 

Fumigation of *Origanum majorana* L. plants with gaseous ozone was conducted during the sixth week after the seeds were sown, in accordance with the method described by Matłok et. al. [23]. Ozone fumigation took place in a plastic chamber described by Szpunar et al. [33]. Subsequently, the aboveground biomass was harvested on the first and fifth day following the treatment, each time from half of the plant pots representing the specific variants of the experiment (duration of ozone treatment). Finally, the raw marjoram leaves were examined for microbial status, total content of polyphenols and antioxidant potential. 

### 3.4. Content of Bioactive Compounds

Fresh plant tissue (2.5 g) was homogenized with 10 ml of 75 % methanol solution using CAT, X-100 homogenizer (Ballr.-Dottingen, Denmark). Next, the homogenate was shaken at room temperature (22–25 °C) for 30 min at 1500 rpm and centrifuged at 7.500× *g* for 30 min. The supernatant obtained was subjected to analysis. The contents of polyphenols in the leaves of *Origanum majorana* L. were measured in accordance with the methodology described by Matłok et at. [33]. Antioxidant potential of marjoram leaves was determined using the ABTS method described by Matłok et al. [33] as well as by the DPPH method described by Oszmiański et al. [34].

### 3.5. Head Space-Solid Phase Microextraction (HS-SPME) and Chromatographic Analysis 

Methodologies described in detail in the Appendix A and Appendix B were followed by head space-solid phase microextraction (HS-SPME) and chromatographic analysis. The analyses were performed in 3 replications.

### 3.6. Microbiological Analysis 

The marjoram leaves were subjected to microbiological tests on the first and the fifth days after the plants were treated with ozone. The enumeration of Lactobacillus spp. in the raw material was determined using the method specified in PN-ISO 15214:2002 “Microbiology of food and animal feeding stuffs—Horizontal method for the enumeration of mesophilic lactic acid bacteria—Colony-count technique at 30 °C”. In accordance with this method, the count of living mesophilic lactic acid bacteria is performed in a solid MRS medium incubated at a temperature of 30 °C for 72 h. The yeast and mould count in 1 g of marjoram was determined using the method described in PN-EN ISO 21527-1:2009 “Microbiology of food and animal feeding stuffs—Horizontal method for the enumeration of yeasts and moulds. Colony count technique at 25 °C.” Enumeration of mesophilic aerobic bacteria in the relevant raw material was performed using the plate count method, in accordance with the standard set forth in (Ae) (O) PB-77/LM, 4th edition dated 07.12.2015. Finally, the presence of endospore producing anaerobes was determined using the method described in (Ae) (O) PB-13/LM, 4th edition, dated 07.04.2014. The analyses were performed in 3 replications. 

### 3.7. Statistical Analysis

To verify the significance of the effect of ozonation time on the quality parameters for each term of analysis, the one-way ANOVA and Tukey post hoc tests were used at α = 0.05. The significance of changes in content of bioactive compounds, microbial load, and chemical composition of headspace fractions during the vegetation time after ozonation was analysed with one-way ANOVA and Tukey post hoc test at α = 0.05. These analyses were performed using STATISTICA12.5 PL from StatSoft.

## 4. Conclusions

*Origanum majorana* L. in the course of production is subject to microbial infections. It was found that ozone treatment effectively reduces aerobic bacteria count, even by 6 log, which considerably increases microbiological safety of plants, and the effect is sustained over time. It was also shown that the potentially labile volatile compounds do not decompose in the conditions of ozone treatment, and their profile remains unchanged. Furthermore, it was established that ozone treatment carried out in an appropriately designed way favourably affects the contents of selected bioactive compounds in the raw material. The best effects were identified in the plants treated with ozone at a rate of 1 ppm for 10 min. It was shown that ozone may be an effective abiotic elicitor which also improves microbiological quality of the raw *Origanum majorana* L. material. The developed method of ozonation of the marjoram plant during the production process is an important factor improving the microbiological quality of the raw material produced. Producing the plant material with appropriate microbiological purity is one of main problem of herb producers. The ozonation method presented in can be successfully implemented in practice and can minimize the problem of microbiological quality of the herbal raw materials produced. In addition, the use of the developed method will also allow for obtaining raw materials with a high content of bioactive compounds, so the produced herbs can be a component of functional food.

## Figures and Tables

**Table 1:** Chemical composition of headspace fractions of *Origanum majorana* L. leaves depending on the time ozonation in one day from ozonation (n = 3).

No.	RT [min]	Peak Share in the Chromatogram (%) Depending on the Time Ozonation						Ordinary Substance Name	Systematic Substance Name	No CAS
		0 min	1 min	3 min	5 min	7 min	10 min			
1	5.13	0.072 ^a^	trace	trace	trace	trace	trace	-	2-hexenal	6728-26-3
2	7.36	1.46 ^b^	2.75 ^c^	0.69 ^a^	1.07 ^b^	2.65 ^c^	1.33 ^b^	α-Pinene	2,6,6-Trimethylbicyclo[3.1.1]hept-2-ene	80-56-8
3	8.35	25.16 ^a^	27.88 ^a^	24.60 ^a^	26.63 ^a^	34.49 ^b^	34.28 ^b^	sabinene	4-methylidene-1-propan-2-ylbicyclo[3.1.0]hexane	3387-41-5
4	8.74	5.40 ^ab^	6.87 ^c^	4.26 ^a^	4.79 ^a^	5.67 ^b^	6.11 ^c^	β-Pinene	2,2,6-trimethyl bicyclo(3.1.1)hept-2-ene	127-91-3
5	9.29	1.79 ^bc^	0.92 ^a^	2.00 ^c^	1.53 ^b^	1.37 ^b^	1.49 ^b^	α-terpinene	1-methyl-4-propan-2-ylcyclohexa-1,3-diene	99-86-5
6	9.44	1.29 ^b^	0.56 ^a^	2.32 ^c^	2.76	1.09 ^b^	1.32 ^b^	α-Phellandrene	2-methyl-5-propan-2-ylcyclohexa-1,3-diene	99-83-2
7	9.54	13.21 ^a^	16.53 ^b^	12.27 ^a^	14.70	12.06 ^a^	12.81 ^a^	3-Carene	3,7,7-trimethylbicyclo[4.1.0]hept-3-ene	13466-78-9
8	10.15	3.07 ^b^	2.77 ^a^	4.30 ^c^	3.16 ^b^	2.35 ^a^	2.58 ^a^	γ-terpinene	1-methyl-4-propan-2-ylcyclohexa-1,4-diene	99-85-4
9	10.31	4.36 ^c^	3.51 ^b^	5.70^d^	4.14 ^c^	2.91 ^a^	2.78 ^a^	sabinene hydrate	(2R,5R)-2-Methyl-5-propan-2-ylbicyclo[3.1.0]hexan-2-ol	17699-16-0
10	10.73	0.91 ^a^	1.56 ^b^	1.25 ^b^	1.05 ^a^	0.74 ^a^	0.80 ^a^	terpinolene	1-methyl-4-propan-2-ylidenecyclohexene	586-62-9
11	10.94	17.00 ^c^	13.76 ^b^	14.07 ^b^	14.47 ^b^	11.83 ^a^	11.74 ^a^	1-terpinenol	1-methyl-4-propan-2-ylcyclohex-3-en-1-ol	586-82-3
12	12.27	0.48 ^b^	0.27 ^a^	1.56 ^d^	0.89 ^c^	0.43 ^b^	0.46 ^b^	4-carvomenthenol	R)-1-Isopropyl-4-methyl-3-cyclohexen-1-ol	562-74-3
13	12.48	0.94 ^b^	2.08 ^d^	1.58 ^c^	1.24	0.65 ^a^	0.78 ^a^	(-)-α-terpineol	2-[(1S)-4-methyl-1-cyclohex-3-enyl]propan-2-ol	10482-56-1
14	13.46	6.86 ^b^	5.68 ^a^	7.77 ^c^	9.01 ^d^	5.77 ^a^	5.40 ^a^	ocimenyl acetate	[(5E)-2,6-dimethylocta-5,7-dien-2-yl] acetate	72214-23-4
15	14.15	0.16 ^b^	0.14 ^b^	trace	trace	0.08 ^a^	0.09 ^a^	terpinyl propionate	2-(4-methyl-1-cyclohex-3-enyl)propan-2-yl propanoate	80-27-3
16	14.71	0.63 ^c^	0.27 ^a^	0.43 ^b^	0.46 ^b^	0.48 ^b^	0.32 ^a^	Bicyclogermacrene	(4E,8E)-4,8,11,11-tetramethylbicyclo[8.1.0]undeca-4,8-diene	100762-46-7
17	14.99	0.20 ^b^	0.33 ^c^	0.33 ^c^	0.25 ^b^	0.15 ^a^	0.15 ^a^	neryl acetate	[(2Z)-3,7-dimethylocta-2,6-dienyl] acetate	141-12-8
18	15.25	0.35 ^c^	0.15 ^a^	0.62 ^e^	0.44 ^d^	0.27 ^b^	0.26 ^b^	lavandulyl acetate	(5-methyl-2-prop-1-en-2-ylhex-4-enyl) acetate	25905-14-0
19	15.91	11.46 ^b^	9.14 ^a^	9.85 ^a^	10.87 ^b^	12.93 ^c^	12.44 ^c^	β-caryophyllene	(−)-trans-Caryophyllene, trans-(1R,9S)-8-Methylene-4,11,11-trimethylbicyclo[7.2.0]undec-4-ene	87-44-5
20	16.33	0.44 ^b^	0.36 ^a^	0.44 ^b^	0.57 ^c^	0.49 ^b^	0.46 ^b^	γ-Elemene	1-ethenyl-1-methyl-2,4-di(propan-2-ylidene)cyclohexane	339154-91-5
TOTAL		95.24 ^b^	95.53 ^b^	94.04 ^a^	98.03 ^d^	96.41 ^c^	95.60 ^b^			

Note: Differences in results between the time of ozonation in current days are indicated by different small letters, with the difference at significant level *p* < 0.05.

**Table 2:** Chemical composition of headspace fractions of leaves of *Origanum majorana* L. leaves depending on the time ozonation in five day from ozonation (n = 3).

No.	RT [min]	Peak Share in the Chromatogram [%] Depending on the Time Ozonation						Ordinary Substance Name	Systematic Substance Name	No CAS
		0 min	1 min	3 min	5 min	7 min	10 min			
1	5.13	0.08 ^a^	trace	trace	trace	trace	trace	-	2-hexenal	6728-26-3
2	7.36	1.34 ^b^	trace	1.18 ^b^	1.39 ^b^	2.39 ^c^	0.66 ^a^	α-Pinene	2,6,6-Trimethylbicyclo[3.1.1]hept-2-ene	80-56-8
3	8.35	26.21 ^b^	23.78 ^a^	23.54 ^a^	25.93 ^b^	26.14 ^b^	23.93 ^a^	sabinene	4-methylidene-1-propan-2-ylbicyclo[3.1.0]hexane	3387-41-5
4	8.74	5.37 ^a^	6.64 ^b^	4.68 ^a^	4.62 ^a^	6.54 ^a^	6.45 ^b^	β-Pinene	2,2,6-trimethyl bicyclo(3.1.1)hept-2-ene	127-91-3
5	9.29	1.97 ^b^	1.16 ^a^	1.34 ^a^	1.04 ^a^	1.74 ^ab^	1.16 ^a^	α-terpinene	1-methyl-4-propan-2-ylcyclohexa-1,3-diene	99-86-5
6	9.44	1.09 ^b^	1.06 ^b^	1.52 ^c^	0.69 ^a^	0.87 ^a^	0.98 ^ab^	α-Phellandrene	2-methyl-5-propan-2-ylcyclohexa-1,3-diene	99-83-2
7	9.54	13.99 ^a^	17.46 ^b^	18.13 ^c^	14.82 ^a^	14.11 ^a^	17.47 ^b^	3-Carene	3,7,7-trimethylbicyclo[4.1.0]hept-3-ene	13466-78-9
8	10.15	3.11 ^b^	2.32 ^a^	2.55 ^ab^	1.90 ^a^	2.97 ^b^	2.04 ^a^	γ-terpinene	1-methyl-4-propan-2-ylcyclohexa-1,4-diene	99-85-4
9	10.31	4.19 ^c^	4.11 ^c^	3.54 ^b^	2.44 ^a^	3.41 ^b^	3.91 ^b^	sabinene hydrate	(2R,5R)-2-Methyl-5-propan-2-ylbicyclo[3.1.0]hexan-2-ol	17699-16-0
10	10.73	0.96 ^b^	0.77 ^a^	0.75 ^a^	0.67 ^a^	0.70 ^a^	0.70 ^a^	terpinolene	1-methyl-4-propan-2-ylidenecyclohexene	586-62-9
11	10.94	16.88 ^b^	15.52 ^a^	19.25 ^c^	16.62 ^b^	15.10 ^a^	15.58 ^a^	1-terpinenol	1-methyl-4-propan-2-ylcyclohex-3-en-1-ol	586-82-3
12	12.27	0.48 ^b^	0.77 ^c^	0.79 ^c^	0.27 ^a^	0.44 ^b^	0.73 ^c^	4-carvomenthenol	R)-1-Isopropyl-4-methyl-3-cyclohexen-1-ol	562-74-3
13	12.48	0.91 ^b^	1.34 ^c^	1.06 ^b^	0.52 ^a^	0.65 ^a^	1.77 ^d^	(-)-α-terpineol	2-[(1S)-4-methyl-1-cyclohex-3-enyl]propan-2-ol	10482-56-1
14	13.46	6.72 ^a^	6.33 ^a^	7.19 ^b^	6.77 ^a^	7.09 ^b^	7.48 ^b^	ocimenyl acetate	[(5E)-2,6-dimethylocta-5,7-dien-2-yl] acetate	72214-23-4
15	14.15	0.19 ^a^	trace	trace	0.07 ^a^	0.44 ^b^	trace	terpinyl propionate	2-(4-methyl-1-cyclohex-3-enyl)propan-2-yl propanoate	80-27-3
16	14.71	0.59 ^a^	0.65 ^a^	0.56 ^a^	1.38 ^b^	1.7 ^bc^	1.95 ^c^	Bicyclogermacrene	(4E,8E)-4,8,11,11-tetramethylbicyclo[8.1.0]undeca-4,8-diene	100762-46-7
17	14.99	0.26 ^b^	0.28 ^b^	0.19 ^a^	0.26 ^b^	0.20 ^a^	0.17 ^a^	neryl acetate	[(2Z)-3,7-dimethylocta-2,6-dienyl] acetate	141-12-8
18	15.25	0.37 ^a^	0.49 ^b^	0.33 ^a^	0.44 ^ab^	0.35 ^a^	0.34 ^a^	lavandulyl acetate	(5-methyl-2-prop-1-en-2-ylhex-4-enyl) acetate	25905-14-0
19	15.91	11.21 ^a^	12.03 ^b^	12.34 ^b^	12.23 ^b^	14.28 ^c^	10.87 ^a^	β-caryophyllene	(−)-trans-Caryophyllene, trans-(1R,9S)-8-Methylene-4,11,11-trimethylbicyclo[7.2.0]undec-4-ene	87-44-5
20	16.33	0.44 ^a^	0.84 ^b^	0.98 ^b^	0.71 ^b^	0.53 ^a^	1.09 ^b^	γ-Elemene	1-ethenyl-1-methyl-2,4-di(propan-2-ylidene)cyclohexane	339154-91-5
TOTAL		96.36 ^c^	95.55 ^b^	99.92 ^d^	92.77 ^a^	99.68	97.28 ^c^			

Note: Differences in results between the time of ozonation in current days are indicated by different small letters, with difference at significant level *p* < 0.05.

**Table 3:** Microbial stress in *Origanum majorana* L. plants on the first and fifth days after ozonation relative to the duration of the treatment (n = 3).

Date of Measurement	Ozonation Time	Count of Aerobic Bacteria (cfu g^−1^)	Count of Yeast and Mould (cfu g^−1^)	Count of Mesophilic Lactic Acid Bacteria (cfu g^−1^)	Presence of Anaerobic Spore Bacteria (cfu g^−1^)
1 day after ozonation	0	7.4 × 10^7 cB^	6.4 × 10^3 cB^	1.2 × 10^2 bA^	absence
	1	5.5 × 10^6 bA^	4.9 × 10^3 cB^	<1.0 × 10^1 aA^	absence
	3	1.8 × 10^6 bA^	1.2 × 10^3 cB^	<1.0 × 10^1 aA^	absence
	5	1.4 × 10^6 bB^	7.7 × 10^2 bB^	<1.0 × 10^1 aA^	absence
	7	5.9 × 10^5 aB^	4.7 × 10^2 bB^	<1.0 × 10^1 aA^	absence
	10	5.5 × 10^1 aB^	<1.0 × 10^1 aA^	<1.0 × 10^1 aA^	absence
5 days after ozonation	0	4.4 × 10^7 dA^	5.5 × 10^3 cA^	1.8 × 10^2 bB^	absence
	1	5.7 × 10^6 cB^	1.6 × 10^3 cA^	<1.0 × 10^1 aA^	absence
	3	1.8 × 10^6 cA^	6.2 × 10^2 bA^	<1.0 × 10^1 aA^	absence
	5	9.8 × 10^5 bA^	5.2 × 10^2 bA^	<1.0 × 10^1 aA^	absence
	7	4.7 × 10^5 bA^	3.2 × 10^2 bA^	<1.0 × 10^1 aA^	absence
	10	6.6 × 10^5 aA^	6.4 × 10^1 aB^	<1.0 × 10^1 aA^	absence

Note: Differences in the results between the time of ozonation in current days are indicated by different small letters, and differences between terms of measurements are indicated by different capital letter, with the difference at significant level *p* < 0.05.

## Appendix A

Head Space–Solid Phase Microextraction HS-SPME: The raw material were subjected to head space–solid phase microextraction analyses, with the use of 100 μm polydimethylsiloxanes (PDMS) fibre manufactured by Supelco Ltd. (Bellefonte, PA, USA). Prior to the analyses, the fibre was conditioned in accordance with the manufacturer’s instruction, at 250 °C for a duration of 30 min in the injector of a gas chromatograph. The refined material, with a weight of 3 ± 0.01 g, was placed in a 100 ml glass vial and sealed with a cap provided with silicone septa. Exposition of the fibre in the headspace phase of the samples took place for 30 seconds at a temperature of 20 °C. Subsequently the fibre was transferred to the injector of the gas chromatograph (temperature 250 °C), where the analytes were thermally desorbed for a duration of 5 min. The composition of the compounds desorbed from SPME fibre was examined using a gas chromatograph (GC-MS, Varian 450GC coupled with 240 MS).

## Appendix B

Chromatographic Analysis: The composition of the desorbed compounds was examined using the gas chromatograph Varian 450 GC with the mass detector (GC-MS) Varian 240 MS (Varian, Palo Alto, California, USA). The carrier gas applied was helium, with a flow rate of 1 ml min−1; the injector temperature was 250 °C. Separation of analytes was conducted using a 30 m × 0.25 mm capillary column, with a moderately polar stationary phase HP-5 (methyl phenyl polysiloxane), with a film thickness of 0.25 μm. The column oven temperature programme at the start was at 50 °C for 5 min of isotherm, followed by an increase of temperature at the rate of 10 °C min−1 up to 300 °C (5 min of isotherm). The analysis was continued for a duration of 35 min. The compounds were identified based on NIST.08 and the Willey database.

## Appendix C

Determination of ozonation process phytotoxicity: At the start, tests were performed to determine the phytotoxicity threshold value; for this purpose, Origanum majorana L. plants were treated with increasing doses of ozone until the phytotoxicity threshold was achieved. The concentration rates of 1, 3, 5, and 10 ppm were applied for 1, 3, 5, 7, and 10 min. The phytotoxicity threshold was examined visually based on the leaves’ necrosis content. Based on this trial, the experimental procedure was proposed.
